# **Cold agglutinin syndrome secondary to Epstein**–**Barr virus reactivation during corticosteroid therapy for minimal change nephrotic syndrome in a patient with human immunodeficiency virus infection**

**DOI:** 10.1007/s13730-026-01145-y

**Published:** 2026-06-22

**Authors:** Harui Bamba, Daisuke Katagiri, Keiki Shimada, Hitomi Otani, Naoto Eriguchi, Yuri Katayama, Mikako Koizumi, Minami Suzuki, Hideki Takano

**Affiliations:** Department of Nephrology, National Center for Global Health and Medicine, Japan Institute for Health Security, 1-21-1 Toyama, Shinjuku-Ku, Tokyo, 162-8655 Japan

**Keywords:** Epstein–Barr virus reactivation, Cold agglutinin syndrome, Minimal change nephrotic syndrome, Human Immunodeficiency Virus infection

## Abstract

Epstein–Barr virus (EBV), a member of the herpesvirus family, establishes lifelong latency following primary infection and may be reactivated under conditions of immunosuppression. EBV reactivation is classically associated with infectious mononucleosis and Burkitt lymphoma and rarely associated with cold agglutinin syndrome. Here, we describe a case of minimal change nephrotic syndrome complicated by cold agglutinin syndrome secondary to EBV reactivation in the setting of human immunodeficiency virus (HIV) infection and corticosteroid-induced immunosuppression. A 63-year-old man with well-controlled HIV infection owing to antiretroviral therapy was admitted for nephrotic syndrome. He was treated with prednisolone (80 mg/day), and renal biopsy performed on day 9 confirmed minimal change nephrotic syndrome. Complete remission was achieved by day 18; however, the patient subsequently developed hyperbilirubinemia, elevated transaminase and lactate dehydrogenase levels, progressive anemia, and thrombocytopenia, accompanied by pharyngitis, malaise, and fever. Laboratory investigations revealed a positive cold agglutinin test, positive direct Coombs test, reduced haptoglobin level, and positive urine urobilinogen, consistent with cold agglutinin syndrome. CMV reactivation was also suggested by transient CMV antigenemia positivity, although no organ-invasive CMV disease was evident. Although EBV serology indicated a past infection, the EBV DNA load was elevated, which suggested viral reactivation. The patient responded to supportive measures, along with early tapering of the corticosteroid dose to reduce immunosuppression. This case highlights that HIV infection in combination with corticosteroid therapy may create an immunosuppressive milieu, predisposing patients to EBV reactivation and subsequent development of cold agglutinin syndrome. Clinicians should be vigilant for EBV-related complications in patients with minimal change nephrotic syndrome requiring corticosteroid therapy, particularly in those with underlying HIV infection.

## Introduction

Epstein–Barr virus (EBV), a member of the herpesvirus family, is a ubiquitous human virus with a global seroprevalence exceeding 90% in adults [1]. Primary EBV infection is often asymptomatic in childhood but may present as infectious mononucleosis in adolescents and young adults. EBV has also been implicated in the pathogenesis of several malignancies, including Burkitt lymphoma, Hodgkin lymphoma, and nasopharyngeal carcinoma [2].

Once established, EBV persists in a latent state within B lymphocytes and can be reactivated in impaired immune surveillance. EBV reactivation has been linked to rare hematological complications, including cold agglutinin syndrome (CAS). Autoimmune hemolytic anemia results from the production of IgM autoantibodies that agglutinate red blood cells at low temperatures, leading to complement-mediated hemolysis.

Immunosuppressive conditions are key predisposing factors for EBV reactivation. Human immunodeficiency virus (HIV), a prototypical cause of chronic immune dysfunction, predisposes patients to a spectrum of renal diseases, including HIV-associated nephropathy and, less frequently, minimal change nephrotic syndrome (MCNS). Furthermore, corticosteroid therapy, which is the mainstay of MCNS treatment, can exacerbate immunosuppression, thereby increasing the risk of viral reactivation.

Herein, we present a case of MCNS complicated by CAS secondary to EBV reactivation in the context of HIV infection and corticosteroid therapy.

## Case report

A 63-year-old man with a history of HIV infection diagnosed 23 years before admission was referred to our department with an 8 kg weight gain over 1 week and bilateral lower extremity edema. His HIV infection was well controlled with antiretroviral therapy (ART) comprising doravirine (100 mg/day), emtricitabine (200 mg/day), and tenofovir alafenamide fumarate (25 mg/day), and the HIV RNA levels were consistently undetectable. Before admission, his CD4 + T-cell count was 793/μL. He also had a history of hepatitis B virus infection and syphilis. Serological testing for syphilis showed a negative rapid plasma reagin test and positive *Treponema pallidum* antibody, consistent with past infection. He had exhibited impaired glucose tolerance (HbA1c levels, 6.1–6.8% for the past 6 years). Urinalysis by dipstick was negative for protein until this admission. His baseline body weight ranged between 85 and 90 kg (a body mass index of approximately 29.5–31.2 kg/m^2^) over the preceding 13 years.

Upon admission, his Glasgow Coma Scale score was E4V5M6. The vital signs were as follows: blood pressure, 154/94 mmHg; heart rate, 77 beats/min; body temperature, 36.6 °C; respiratory rate, 18 breaths/min; and oxygen saturation, 95% on room air. His body weight was 95.3 kg (body mass index of 33.1 kg/m^2^), representing an 8 kg increase from the baseline. Physical examination revealed bilateral pitting edema of the lower extremities, without periorbital edema. Cardiopulmonary auscultation results were unremarkable.

The initial laboratory findings (Table 1) demonstrated hypoalbuminemia, hyperlipidemia, and heavy proteinuria, fulfilling the diagnostic criteria for nephrotic syndrome, along with mild hyperglycemia and hypercoagulability. Immunological studies revealed low immunoglobulin (Ig)G and elevated IgA levels, whereas other parameters were within normal limits. Serological testing was negative for hepatitis B viral DNA, and CD4 + T-cell counts were preserved. Abdominal computed tomography revealed ascites but no renal morphological abnormalities or mass lesions suggestive of malignancy.

**Table 1:** Laboratory findings on admission

	On admission	Normal range
White blood cell (/μL)	5060	3300–8600
Neutrophil (%)	54.1	39.6–67.0
Lymphocytes (%)	35.4	24.0–48.4
Erythrocyte (× 10^6^/μL)	5.4	4.4–5.6
Hemoglobin (g/dL)	17.2	13.7–16.8
Platelet (× 10^3^/μL)	23.1	15.8–34.8
Total protein (g/dL)	4.8	6.6–8.1
Albumin (g/dL)	1.3	4.1–5.1
Urea (mg/dL)	18	8–20
Creatinine (mg/dL)	1.35	0.65–1.07
Aspartate aminotransferase (IU/L)	39	13–30
Alanine aminotransferase (IU/L)	33	10–42
Total bilirubin (mg/dL)	0.3	0.4–1.5
Lactate dehydrogenase (IU/L)	221	124–222
Alkaline phosphatase (U/L)	84	38–113
Sodium (mEq/L)	142	138–145
Potassium (mEq/L)	4.7	3.6–4.8
Chloride (mEq/L)	107	101–108
C-reactive protein (mg/dL)	0.11	0.00–0.14
Triglyceride (mg/dL)	522	40–149
High-density lipoprotein cholesterol (mg/dL)	44	39–90
Low-density lipoprotein cholesterol (mg/dL)	208	65–139
Fasting blood sugar (g/dL)	138	80–110
Hemoglobin A1c (%)	6.2	4.9–6.0
Prothrombin time (%)	115	80–100
Activated partial thromboplastin time (s)	31	25–35
Fibrinogen (mg/dL)	685	200–400
D dimers (μg/mL)	4.3	0.0–1.0
Hematuria	1 +	Negative
Proteinuria	4 +	Negative
Urinary leukocytes (/HPF)	5–9	< 10
Urinary erythrocytes (/HPF)	5–9	< 5
Urinary protein (g/day)	18.2	0.02–0.12
Hepatitis B surface antigen (IU/mL)	36.8	Negative
Hepatitis B virus DNA	Negative	Negative
Hepatitis C virus antibody	Negative	Negative
CD4^+^ T-cell (/μL)	793	500–1200
Immunoglobulin G (mg/dL)	687	861–1747
Immunoglobulin A (mg/dL)	418	93–393
Immunoglobulin M (mg/dL)	75	33–183
Complement C3 (U/L)	133	73–138
Complement C4 (mg/dL)	33	11–31
Antinuclear antibody (times)	< 40	< 40
Myeloperoxidase anti-neutrophil cytoplasmic antibody	Negative	Negative
Proteinase 3 anti-neutrophil cytoplasmic antibody	Negative	Negative
Anti-glomerular basement membrane antibody	Negative	Negative
Cryoglobulin	Negative	Negative

**Fig. 1:**
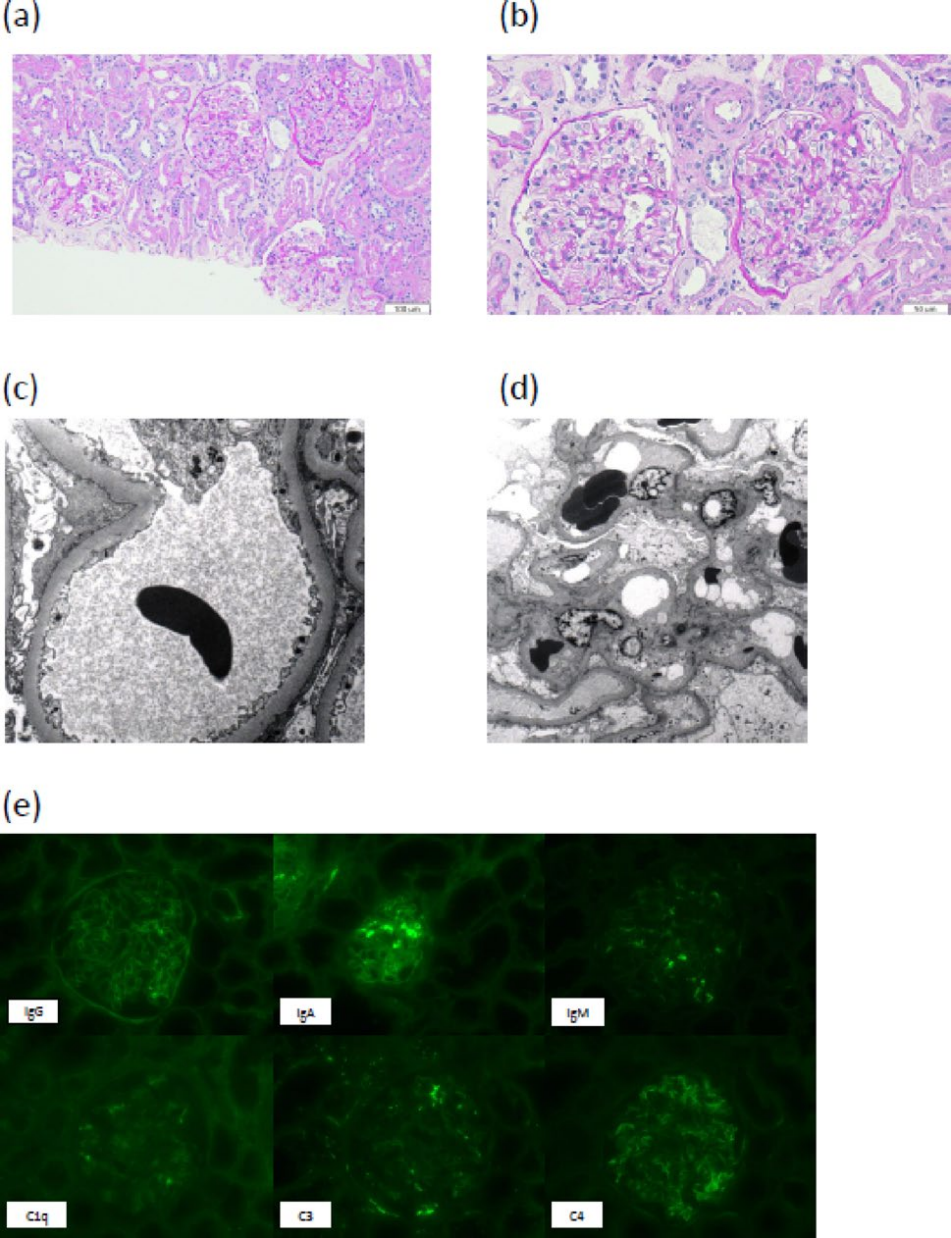
Pathological findings of renal biopsy, a. Light microscopy shows partial glomerular hypertrophy and a slight increase in mesangial matrix (periodic acid–Schiff staining; scale bar = 100 μm), b. Higher magnification of the same glomerulus highlights the mild mesangial matrix expansion (periodic acid–Schiff staining; scale bar = 50 μm), c. Electron microscopy reveals foot process effacement and basement membrane thickening (original magnification × 5000), d. Electron microscopy of a glomerular area including the mesangium demonstrates no electron dense deposits in any portion of the glomerulus (original magnification × 1500), e. Immunofluorescence studies reveal mild deposition of IgA, IgM, C3, and C4 in the mesangial area

Given the severity of nephrotic syndrome and suspected impaired absorption of oral agents due to intestinal edema, treatment was initiated on day 1 of hospitalization with intravenous water-soluble prednisolone at 80 mg/day (equivalent to oral prednisolone at 60 mg/day). The patient was administered intravenous albumin and furosemide for volume management. Renal biopsy was performed on day 9 of hospitalization (Fig. 1). Light microscopy showed mild mesangial expansion and glomerular hypertrophy but no cystic lesions typical of HIV-associated nephropathy. Immunofluorescence microscopy revealed weak IgA deposition in the mesangial areas, along with trace IgM and C3 deposits. Electron microscopy revealed diffuse effacement of the podocyte foot processes and focal thickening of the glomerular basement membrane without electron-dense deposits. These findings were consistent with those of MCNS, although the concomitant effects of diabetes mellitus and obesity could not be excluded. No pathological findings suggestive of hemolysis were identified in the biopsy specimen; however, given the limited sampling nature of renal biopsy, focal changes could not be completely excluded.

By day 12 of hospitalization, proteinuria and fluid overload began to improve, allowing the transition to oral prednisolone at 60 mg/day and subsequent tapering of the dose to 50 mg/day by day 15.

However, on day 11, progressive hyperbilirubinemia and elevated lactate dehydrogenase (LDH) levels were observed, followed by an increase in transaminase levels from day 15 onward. Simultaneously, hemoglobin levels and platelet counts began to decline, accompanied by pharyngitis, malaise, and fever. By day 19, hemoglobin levels decreased to 5.6 g/dL, and the platelet count was 38,000/μL, necessitating multiple red blood cell transfusions. On the same day, laboratory tests revealed a total bilirubin level of 2.5 mg/dL (indirect bilirubin, 1.8 mg/dL), aspartate aminotransferase level of 36 U/L, alanine aminotransferase level of 44 U/L, and LDH level of 463 U/L, prompting further investigation of hemolysis (Table 2).

**Table 2:** Laboratory findings during the evaluation of liver dysfunction and cytopenia

	Day 19	Normal range
White blood cell (/μL)	14,900	3300–8600
Neutrophil (%)	83.5	39.6–67.0
Lymphocytes (%)	8.0	24.0–48.4
Erythrocyte (× 10^6^/μL)	1.5	4.4–5.6
Hemoglobin (g/dL)	5.6	13.7–16.8
Reticulocyte (%)	10.23	0.82–2.25
Platelet (× 10^3^/μL)	3.8	15.8–34.8
Aspartate aminotransferase (IU/L)	36	13–30
Alanine aminotransferase (IU/L)	44	10–42
Total bilirubin (mg/dL)	2.5	0.4–1.5
Indirect bilirubin (mg/dL)	1.8	≤ 0.8
Lactate dehydrogenase (IU/L)	463	124–222
Alkaline phosphatase (U/L)	141	38–113
C-reactive protein (mg/dL)	1.88	0.00–0.14
Haptoglobin (mg/dL)	< 10	19–170
Cold agglutinin (times)	> 1024	≤ 63
Direct Coombs test	Positive	Negative
Hematuria	Negative	Negative
Proteinuria	±	Negative
Urobilinuria	1 +	Negative

Hematological evaluation revealed indirect hyperbilirubinemia, reduced haptoglobin levels, reticulocytosis, positive urine urobilinogen, and positive direct Coombs test (IgG negative, C3d positive). The cold agglutinin test yielded positive results, and a peripheral smear revealed platelet agglutination. Notably, the platelet count measured in the citrate-anticoagulated sample was within normal limits (167,000/μL), suggesting pseudothrombocytopenia due to in vitro platelet aggregation. Collectively, these findings confirmed the diagnosis of CAS, a subtype of autoimmune hemolytic anemia.

Evaluation of the underlying etiologies of CAS (Table 3) revealed EBV serology consistent with a past infection (viral capsid antigen IgG positive, viral capsid antigen IgM negative, and Epstein–Barr nuclear antigen positive). However, quantitative polymerase chain reaction revealed elevated EBV DNA levels, suggesting viral reactivation. Although soluble interleukin-2 receptor levels were slightly elevated, computed tomography revealed no lymphadenopathy or masses, indicating that lymphoma was unlikely. Screening for Mycoplasma pneumoniae infection yielded negative results. CMV antigenemia was slightly positive on day 18 (0 cells/slide 1 and 1 cell/slide 2) and increased on day 26 (2 cells/slide 1 and 3 cells/slide 2), but subsequently became negative by day 36. In addition, CMV IgM increased to 12.65 (index) on day 40. However, there was no clinical evidence of organ-invasive CMV disease. Therefore, EBV reactivation was considered the most likely trigger of CAS in this patient, although concomitant CMV reactivation could not be completely excluded.

**Table 3:** Laboratory findings during the evaluation of the cause of cold agglutinin syndrome

	Day 19	Normal range
Hepatitis A virus antibody – IgG	Negative	Negative
Hepatitis A virus antibody – IgM	Negative	Negative
Hepatitis B virus DNA	Negative	Negative
Hepatitis E virus antibody – IgA	Negative	Negative
Cytomegalovirus antibody – IgG (AU/mL)	215.1	6.0
Cytomegalovirus antibody – IgM (Index)	0.93	0.85
CMV antigenemia (pp65) (cells/slide)	0/1*	Negative
Epstein**–**Barr virus, viral capsid antigen – IgG (times)	640	10
Epstein**–**Barr virus, viral capsid antigen – IgM (times)	< 10	10
Epstein**–**Barr virus, nuclear antigen (times)	80	10
Epstein-Barr virus DNA (copy/mL)	20,000	< 200
*Mycoplasma pneumoniae* DNA	Negative	Negative
Soluble interleukin-2 receptor (U/mL)	1441	204–587

^*^0/1 indicates 0 cells in slide 1 and 1 cell in slide 2

**Fig. 2:**
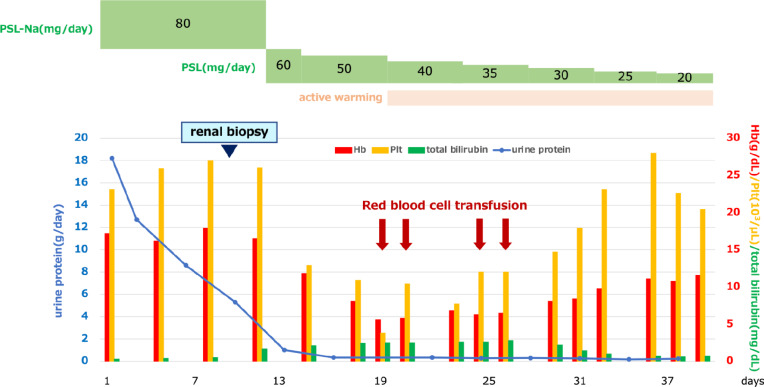
Clinical course of the case. The CD4 + T-cell counts were 793/μL at admission and 759/μL on day 40 of hospitalization. Abbreviations: PSL-Na, prednisolone sodium succinate; PSL, prednisolone; Hb, hemoglobin; Plt, platelet

Supportive care included maintenance of normothermia and repeated red blood cell transfusions. Given the suspected role of corticosteroid-induced immunosuppression in EBV reactivation, the prednisolone dose was tapered more rapidly than originally planned. Despite steroid reduction, the patient maintained complete remission of MCNS. On day 28 of hospitalization, the prednisolone dose was tapered to 30 mg/day, with subsequent improvement in CAS. Further dose tapering to 20 mg/day was achieved, and the patient was discharged on day 41 in a stable condition (Fig. 2). At follow-up after discharge (day 47), EBV DNA became undetectable.

## Discussion

HIV infection is associated with a wide spectrum of renal complications that affect the glomeruli, tubulointerstitium, and vasculature. The most well-characterized entity is HIV-associated nephropathy, which is characterized by collapsing focal segmental glomerulosclerosis, microcystic tubular dilatation, and interstitial inflammation [3]. MCNS is rare in this population, accounting for only 0.7–1.05% of cases [4, 5]. In the present case, no collapsing lesions suggestive of HIV-associated nephropathy were observed on light microscopy. Moreover, electron microscopy revealed diffuse foot process effacement without electron-dense deposits, making HIV immune complex kidney disease unlikely. Although HIV infection may theoretically contribute to secondary MCNS development through immune dysregulation, our patient had well-controlled HIV infection at the onset of nephrotic syndrome, with a CD4 + T-cell count of 793/μL and an undetectable viral load. Therefore, the findings are mostly consistent with idiopathic MCNS occurring in well-controlled HIV infection.

Previous case reports of MCNS in patients with HIV infection have demonstrated favorable outcomes with standard corticosteroid therapy, typically prednisolone at 1 mg/kg/day [6, 7]. The 2014 Infectious Diseases Society of America guidelines recommend corticosteroids as a weak adjunctive option for biopsy-proven HIV-associated nephropathy, in combination with ART and renin–angiotensin system blockade [8]. Importantly, randomized trials have suggested that corticosteroid administration does not significantly affect CD4 + T-cell counts or plasma HIV RNA levels [9]. Nonetheless, concerns remain regarding the potential risks, including ischemic necrosis and opportunistic infections [8].

In this case, HIV infection was well controlled with ART, with a CD4 + T-cell count of 793/μL. Considering the patient’s body weight of 95.3 kg (an 8 kg increase due to edema), corticosteroid therapy was initiated with intravenous water-soluble prednisolone at 80 mg/day, consistent with the conventional treatment for MCNS.

Despite remission of proteinuria with corticosteroid therapy, the patient developed hyperbilirubinemia, elevated LDH and transaminase levels, progressive anemia, and thrombocytopenia. The workup confirmed CAS, which is a subtype of autoimmune hemolytic anemia. Cold agglutinins are predominantly IgM autoantibodies that bind to red blood cells at low temperatures (0–4 °C), causing agglutination and complement activation [10, 11]. Complement-mediated hemolysis primarily occurs via the extravascular clearance of C3b-opsonized erythrocytes by hepatic Kupffer cells, although severe intravascular hemolysis can occur when complement activation leads to the formation of a membrane attack complex.

Cold agglutinin disease (CAD) is categorized as a primary or secondary disease. The term CAS has been proposed for cases secondary to underlying conditions, such as infections or malignancies [12]. The reported infectious triggers include *Mycoplasma pneumoniae*, EBV, cytomegalovirus, and severe acute respiratory syndrome coronavirus 2 [13–15]. In our case, CMV reactivation was also suggested by transient CMV antigenemia positivity and an elevated CMV IgM level. However, there was no evidence of organ-invasive CMV disease. Therefore, the patient was managed with careful monitoring and early tapering of corticosteroids. The clinical manifestations of primary CAD and secondary CAS are generally similar and primarily derived from hemolysis and peripheral circulatory disturbances. However, cold-induced peripheral circulatory symptoms may be minimal in warm environments or in the absence of significant cold exposure. CAS secondary to infections typically presents acutely and is often accompanied by hemoglobinuria and severe anemia. In cases associated with EBV infection, symptoms usually emerge 2–3 weeks after the onset of infection, and hemolysis resolves within approximately 2 weeks [16]. Peripheral circulatory symptoms may include cyanosis of the acral regions (e.g., digits, nasal tip, and auricles), sensory disturbances, and Raynaud-like phenomena, which are attributed to the agglutination of erythrocytes within the dermal microvasculature [12]. In this case, the onset occurred during the summer months, and the absence of cold exposure likely contributed to the minimal manifestation of circulatory symptoms.

Further evaluation revealed EBV serology consistent with a past infection; however, EBV DNA levels were elevated, suggesting viral reactivation. In our case, EBV DNA became undetectable after clinical recovery (day 47), supporting clinically relevant EBV reactivation rather than incidental viremia. Cold agglutinins are detected in up to 60% of patients with acute EBV infection; however, only 0.5–3% of them develop clinically significant hemolysis [17, 18]. EBV persists for a lifetime in latently infected B lymphocytes and is usually controlled by CD4 + and CD8 + T-cell–mediated immune surveillance. However, immunosuppression can lead to viral reactivation and clinical sequelae [19]. Although corticosteroid-induced immunosuppression can facilitate reactivation of latent EBV, the precise interval between initiation of immunosuppressive therapy and viral reactivation or subsequent manifestations, such as CAS, remains unclear. In our patient, CAS developed approximately 11 days after starting steroids, a timeframe that is within a plausible range given the complexity of host–virus interactions under immunosuppressive conditions.

Individuals with HIV harbor 10–20 times more EBV-infected B cells than healthy controls, and their T-cell responses are insufficient to suppress viral proliferation [20]. EBV reactivation has been reported to cause diverse complications in patients with HIV, including hemophagocytic lymphohistiocytosis and acute liver failure [21, 22]. Corticosteroid therapy has also been implicated as a potential trigger for EBV reactivation [23]. In this case, both HIV-associated immune dysregulation and corticosteroid-induced immunosuppression likely contributed to EBV reactivation, which subsequently triggered CAS. In HIV-infected individuals, EBV-associated B-cell lymphoproliferative disorders (LPDs) should be considered in the differential diagnosis, as they may be associated with secondary CAS. However, in our patient, plain computed tomography showed no lymphadenopathy, although soluble interleukin-2 receptor levels were slightly elevated. Furthermore, the hemolysis followed an acute and reversible course, which is atypical of CAS associated with clonal B-cell disorders. These findings make EBV-associated LPD unlikely and instead indicate transient immune dysregulation due to EBV reactivation.

The patient responded to supportive measures, including maintenance of normothermia and transfusion, along with early tapering of the corticosteroid dose to reduce immunosuppression. Steroid reduction did not compromise proteinuria remission, and both MCNS and CAS improved with this strategy. The management of CAS remains largely empirical, as no randomized trials have validated specific interventions [24]. Corticosteroids have limited efficacy in CAD/CAS and are not generally recommended [13, 25]. Therefore, treating the underlying trigger is crucial in secondary cases. Although EBV reactivation was considered the most plausible trigger based on the elevated EBV DNA level and compatible systemic symptoms, concomitant CMV reactivation was also suggested by transient CMV antigenemia positivity and elevated CMV IgM levels. Therefore, a contributory role of CMV cannot be completely excluded.

To our knowledge, this is the first reported case of EBV reactivation-associated CAS developing during corticosteroid therapy in a patient with HIV infection. In the present case, corticosteroid treatment for MCNS preceded the onset of CAS. This case highlights the need for careful monitoring of EBV-related complications when initiating corticosteroids in immunocompromised patients.
